# Supplementary figures and images for: The molecular targets of ivermectin and lotilaner in the human louse Pediculus humanus humanus: New prospects for the treatment of pediculosis
Source: PLoS Pathog. 2021 Feb 18;17(2):e1008863. doi: 10.1371/journal.ppat.1008863 (PMC7891696; doi:10.1371/journal.ppat.1008863)

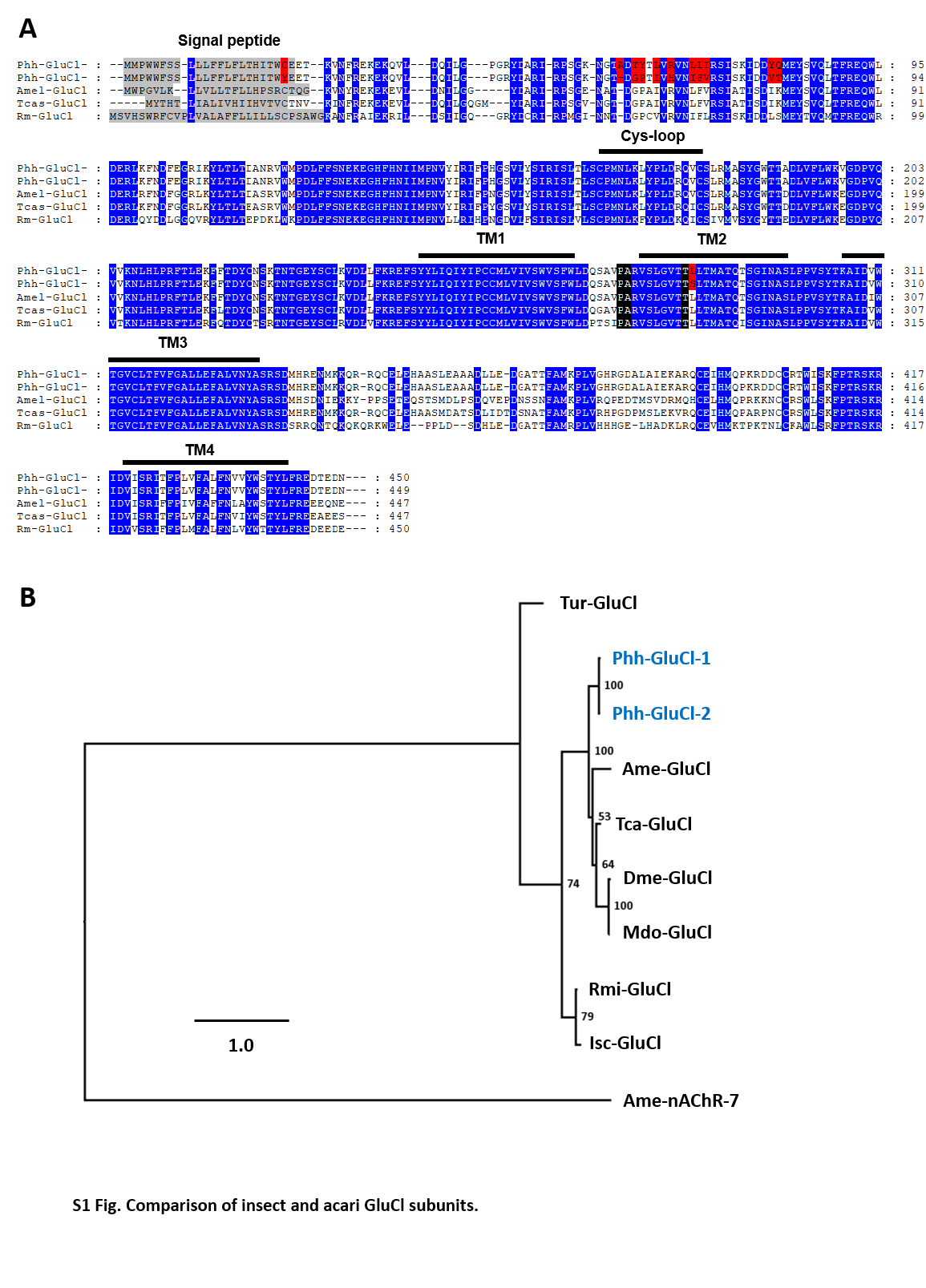

Supplement: S1 Fig — A. Alignment of GluCl subunit deduced amino-acid sequences from Pediculus humanus humanus (Phh), Apis mellifera (Ame), Rhipicephalus microplus (Rmi) and Tribolium castaneum (Tca). Predicted signal peptides in N-terminal are highlighted in grey. Amino acid differences between the GluCl-1 and GluCl-2 sequences of P. humanus humanus are highlighted in red. Amino acids conserved between all the sequences are highlighted in blue. The cys-loop, transmembrane domains (TM1-TM4) and the highly variable intracellular loop are indicated by the bars. B. Distance tree (BioNJ, Poisson) of GluCl protein sequences from insects and acari. The three letter prefixes in gene names Tur, Phh, Ame, Tca, Dme, Mdo, Rmi and Isc refer to the species Tetranychus urticae, Pediculus humanus humanus, Apis mellifera, Tribolium castaneum, Drosophila melanogaster, Musca domestica, Rhipicephalus microplus and Ixodes scapularis, respectively. The tree was rooted with the A. mellifera alpha7 nAChR subunit as an outgroup. Branch lengths are proportional to the number of substitutions per amino acid. Scale bar represents the number of substitutions per site. The bootstrap values are indicated next to each branch. Accession numbers for sequences used in the phylogenetic analysis are provided in the Methods section. The two GluCl sequences of interest are highlighted in blue. (TIF) [file ppat.1008863.s001.tif]

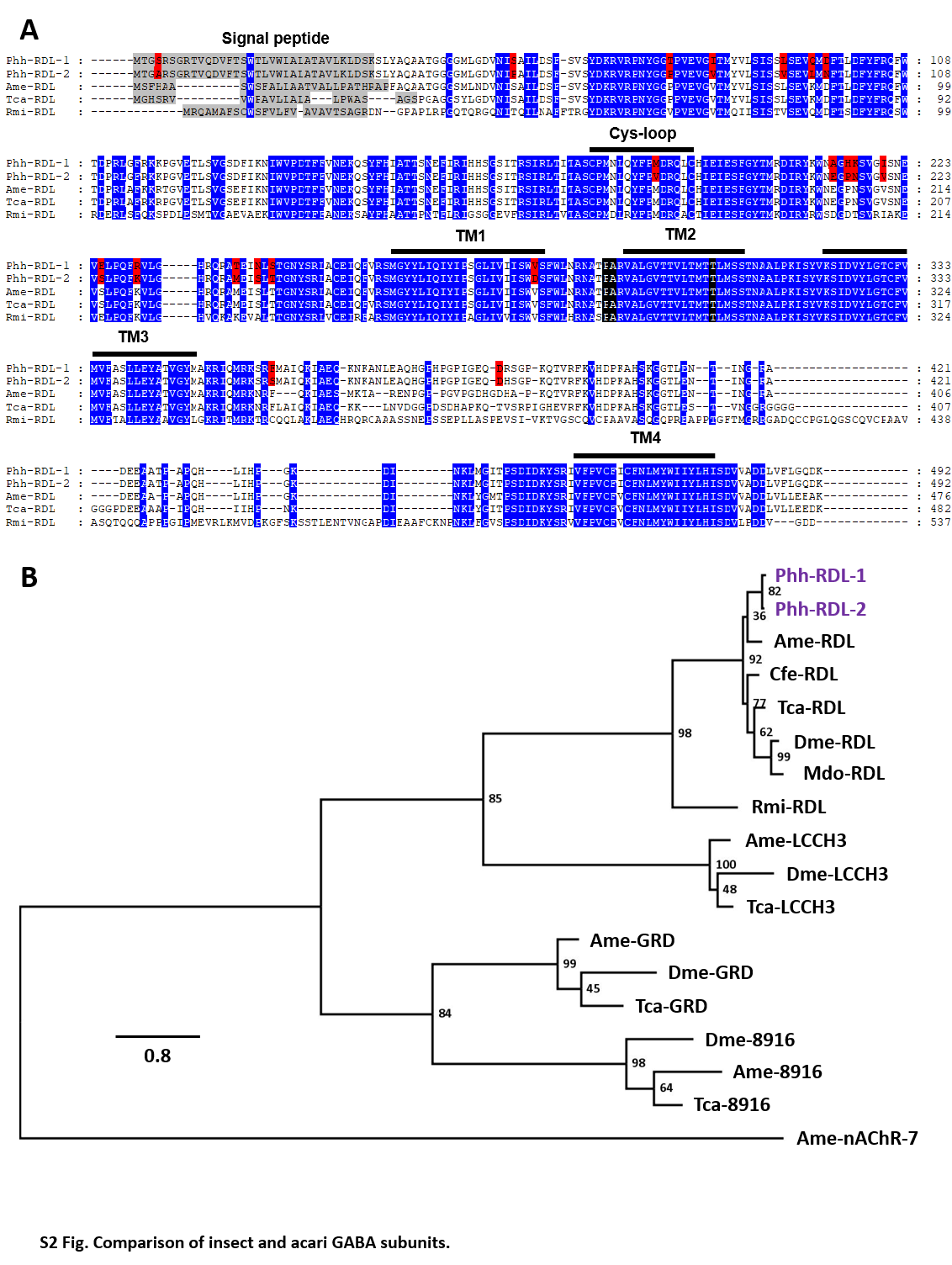

Supplement: S2 Fig — A. Alignment of RDL subunit deduced amino-acid sequences from Pediculus humanus humanus (Phh), Apis mellifera (Ame), Tribolium castaneum (Tca) and Rhipicephalus microplus (Rmi). Predicted signal peptides in N-terminal are highlighted in grey. Amino acid difference between the RDL-1 and RDL-2 sequences of P. humanus humanus are highlighted in red. Amino acids conserved between all the sequences are highlighted in blue. The cys-loop, predicted transmembrane domains (TM1-TM4) and the highly variable intracellular loop are indicated by the bars. B. Distance tree (BioNJ, Poisson) of GABACl protein sequence from insects and acari. The three letter prefixes in gene names Phh, Ame, Cfe, Tca, Dme, Mdo and Rmi refer to the species Pediculus humanus humanus, Apis mellifera, Ctenocephalides felis, Tribolium castaneum, Drosophila melanogaster, Musca domestica and Rhipicephalus microplus, respectively. The tree was rooted with the A. mellifera alpha7 nAChR subunit as an outgroup. Branch lengths are proportional to the number of substitutions per amino acid. Scale bar represents the number of substitutions per site. The bootstrap values are indicated next to each branch. Accession numbers for sequences used in the phylogenetic analysis are provided in the Methods section. The two RDL sequences of interest are highlighted in purple. (TIF) [file ppat.1008863.s002.tif]

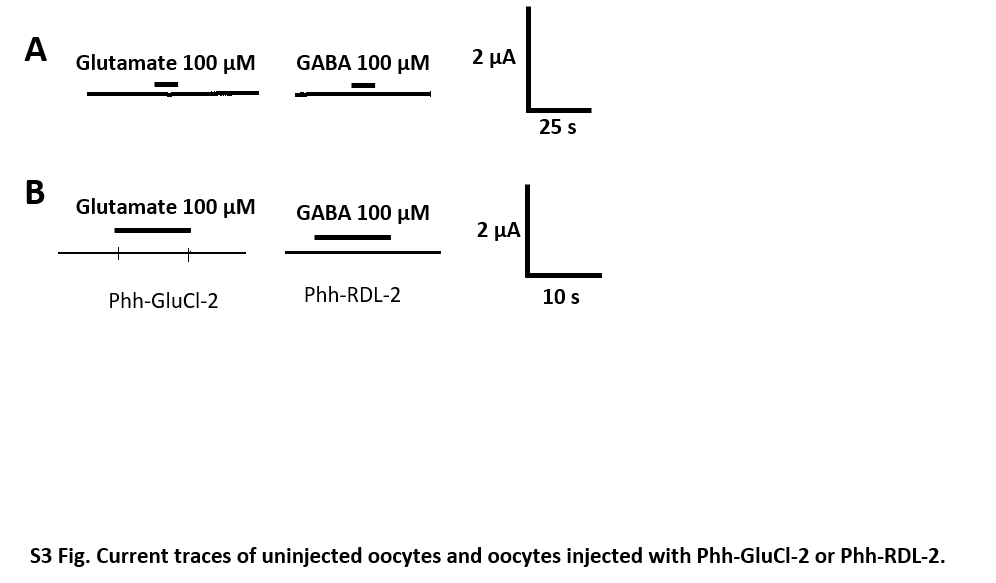

Supplement: S3 Fig — A. Current traces of uninjected oocytes after application of glutamate and GABA at 100 μM. The bar indicates the application time of 10 s. B. For Phh-GluCl-2, oocytes were clamped at -80mV and response to 100 μM of glutamate was measured (n = 11). For Phh-RDL-2, oocytes were clamped at -60mV and response to 100 μM of GABA was measured (n = 12). The bar indicates the application time of 10 s. (TIF) [file ppat.1008863.s003.tif]

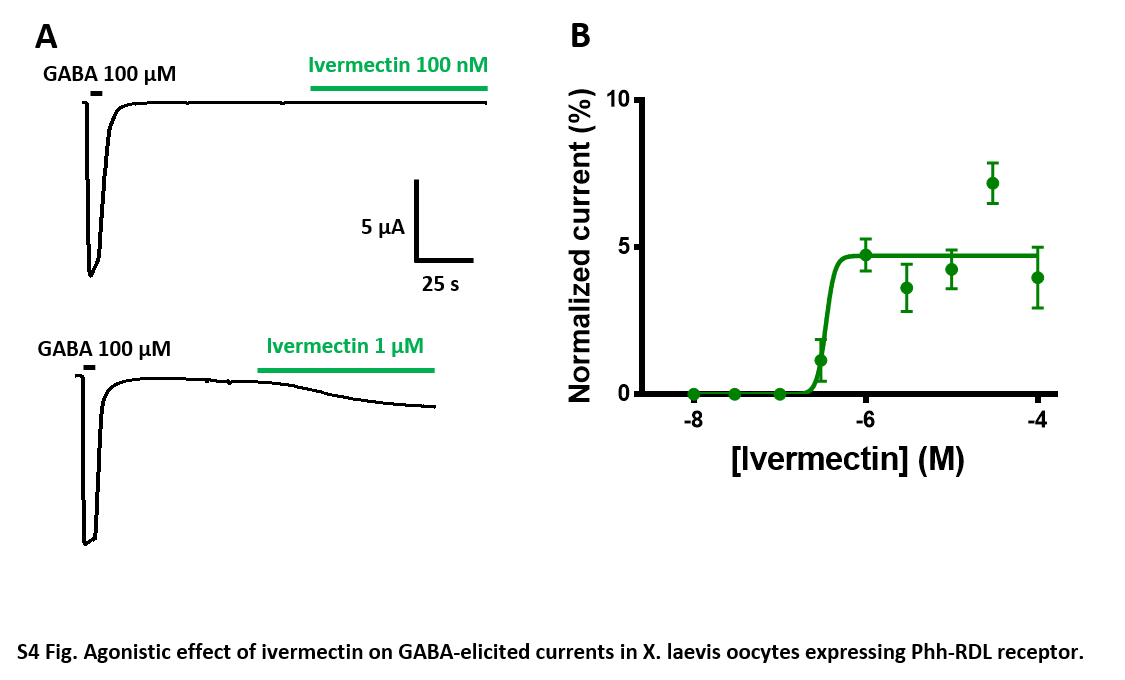

Supplement: S4 Fig — A. Representative current traces evoked by 100 μM GABA followed by 90 s application of 100 nM or 1 μM ivermectin. Application times are indicated by the bars. B. Concentration response curve of the ivermectin agonist effect. Data are normalized to a first 100 μM GABA application (mean +/- SEM, n = 5–13). (TIF) [file ppat.1008863.s004.tif]

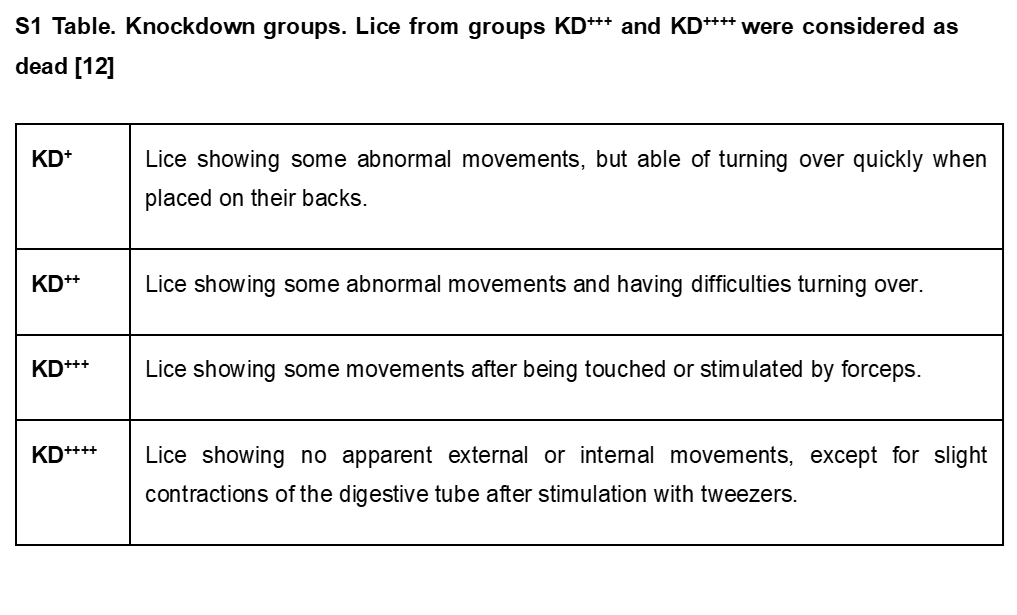

Supplement: S1 Table — (TIF) [file ppat.1008863.s005.tif]

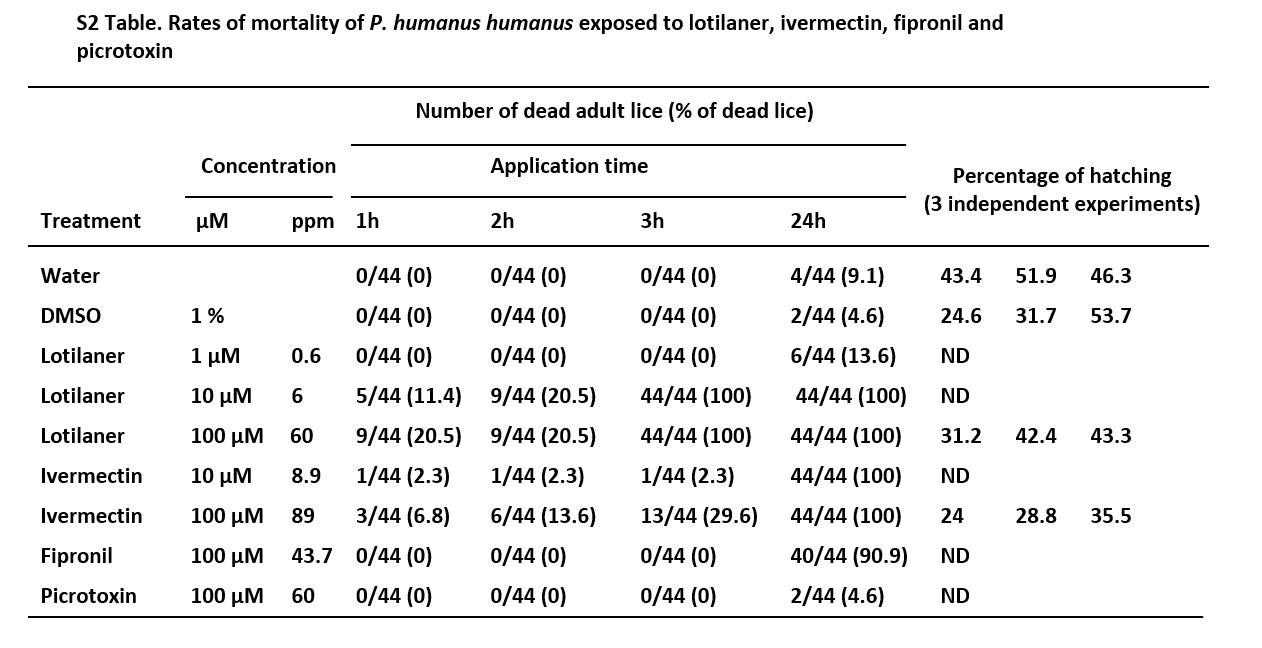

Supplement: S2 Table — (TIF) [file ppat.1008863.s006.tif]

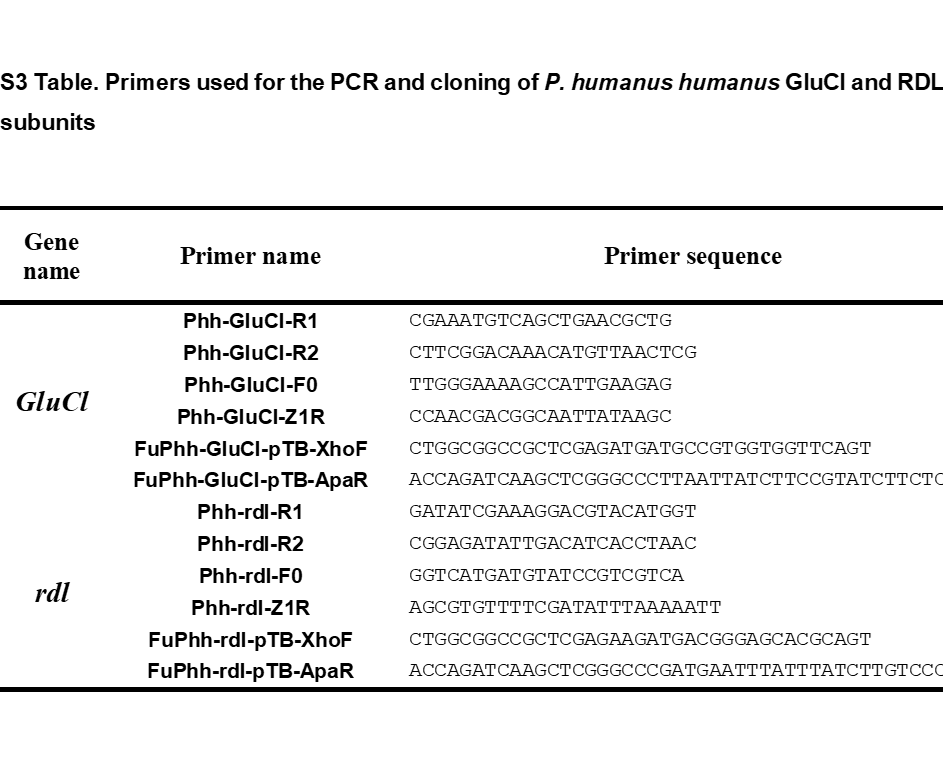

Supplement: S3 Table — (TIF) [file ppat.1008863.s007.tif]
